# Hepatotoxicity Assessment of Anshenbunao Syrup by Multi-Component Quantification In Vivo/In Vitro and Cell Biological Evaluations

**DOI:** 10.3390/ph19030404

**Published:** 2026-03-01

**Authors:** Lan Chen, Zhizhen Wei, Rui Cheng, Pengwei Hu, Shixiao Wang, Wei Wu, Adouani Imene, Yuan Zhang, Fengming Chen, Taijun Hang

**Affiliations:** 1 Department of Pharmaceutical Analysis, China Pharmaceutical University, Nanjing 210009, China; 2 School of Pharmacy, Shandong Second Medical University, Weifang 261053, China; 3 Department of Pharmacy, Faculty of Medicine, University of Ferhat Abbas Setif 1, Setif 19000, Algeria; 4 School of Pharmacy, China Medical University, Shenyang 110122, China; 13840149878@163.com; 5 Institute of Food Safety, Chinese Academy of Quality and Inspection & Testing, Beijing 100176, China

**Keywords:** Anshenbunao syrup, pharmacokinetics, chemical basis, drug-induced liver injury

## Abstract

**Background/Objectives:** There is high demand for Anshenbunao syrup (ABS) in Chinese medicine owing to its steady therapeutic efficacy for insomnia and neurasthenia. However, it contains a substantial proportion of *Polygoni Multiflori Radix Praeparata* (PMRP), which is associated with reported cases of drug-induced liver injury (DILI). Here, we aim to establish an integrated approach combining PK screening with a dual-model toxicity verification system to systematically identify liver injury components (from high to low concentrations and from direct to idiosyncratic hepatotoxicity) to accurately uncover diverse potential hepatotoxicity markers. **Methods:** A sensitive UPLC-MS/MS method was used to accurately quantify the components in plasma at the ng/mL level and conduct a pharmacokinetic analysis. Rat models were used to evaluate exposure levels of the eight active constituents and three major metabolites after a single oral gavage dose of 10 mL/kg ABS and identify the quality markers. The early-stage and high-throughput assessment of direct and idiosyncratic hepatotoxicity was conducted in vitro utilizing HepG2 cells. After the administration of the quality markers (0.01–80 μM), CCK-8 was used to detect cell viability on both normal and susceptible cells, and the latter was induced by lipopolysaccharide. **Results:** As a result, seven quality markers were screened based on their contents and exposure levels in rat plasma by UPLC–MS/MS, including emodin (EM), liquiritin (LI), 2,3,5,4′–Tetrahydroxystilbene–2–*O*–*β*–*D*–glucoside (TSG), icariin, emodin–8–*O*–*β*–*D*–glucoside, baohuoside I (BA), and 18*β*–glycyrrhetinic acid (GTA). Moreover, the half maximal inhibitory concentration values of both normal cells and the lipopolysaccharide-induced immune stress liver injury cells were fitted within the concentration range of 0.01–80 μM, based on which, EM, BA, and GTA were identified as the principal hepatotoxic constituents in ABS at elevated concentrations. This study is the first to demonstrate that TSG, EM, LI, and GTA exhibit synergistic cytotoxicity in LPS-sensitized hepatocytes at clinically relevant concentrations, whereas EM was also a direct hepatotoxic component. Given that TSG is one of the major ingredients in ABS, the underappreciated idiosyncratic hepatotoxicity could elevate the risk of adverse clinical outcomes. **Conclusions:** In conclusion, this study effectively identifies hepatotoxic constituents in ABS and evaluates their hazards under immune stress and toxicity profiles in clinical concentrations, which also provides a robust foundation for the awareness of PMRP-induced DILI due to ABS.

## 1. Introduction

*Polygoni Multiflori Radix Praeparata* (PMRP), which is the processed form of *Polygoni Multiflori Radix* (PMR), is the key botanical material in the Anshenbunao syrup (ABS) prescription. The Chinese Pharmacopoeia includes 53 traditional Chinese medicines (TCMs) containing PMRP (6–12 g/day), among them ABS stands out as one of the most famous formulations with clinically validated therapeutic benefits as it can ameliorate cognitive dysfunction and attenuate neuronal degeneration [1]. However, the increasing incidence of herb-induced liver injury has heightened public concern regarding the safety of herbal products [2,3,4]. Herbal products are most frequently associated with adverse reactions involving hepatotoxicity [5], and the intricate chemical composition of ABS also poses additional challenges for the assessment of its hepatotoxicity.

The central challenge in assessing the safety of PMRP-containing formulations like ABS lies not merely in their complex phytochemical profiles, but in the fundamental disconnect between preclinical safety predictions and clinical reality—a gap that remains inadequately addressed by current hepatotoxicity assessment standards [6,7,8]. While risk factors for herb-induced liver injury are broadly categorized into herb-related (e.g., processing quality), patient-specific (e.g., metabolic enzyme activity), and prescription-related (e.g., dosing) domains, these factors alone fail to explain the sporadic, idiosyncratic nature of liver injury reports associated with PMRP products administered at recommended doses [9,10]. This paradox—well-documented efficacy at safe doses versus unpredictable, low-incidence toxicity [11,12]—represents the critical unmet need driving this study: the absence of a mechanistic framework capable of identifying which constituents trigger hepatotoxicity in susceptible individuals, under what conditions, and through which pathways. Given this, the precise quantification and evaluation of various constituents in ABS, both in terms of in vivo/in vitro concentrations and biological activities, hold substantial scientific and clinical significance.

2,3,5,4′–Tetrahydroxystilbene–2–*O*–*β*–*D*–glucoside (TSG) and emodin (EM) are the major bioactive constituents of PMRP. TSG demonstrates cardioprotective, melanogenic, and anti-aging properties [13,14,15,16], while EM exhibits both anti-cancer activity [17] and pronounced hepatotoxicity through mechanisms involving ferroptosis, cholestasis, and glutathione depletion [18,19]. Moreover, the anthraquinone glycosides, such as emodin–8–*O*–*β*–*D*–glucoside (EMG), in processed PMRP undergo hydrolysis into their corresponding aglycones (e.g., EM) in the gastrointestinal tract, thereby enhancing their systemic bioavailability [19,20]. Thence, the collective toxicological effects of ABS components and their pharmacokinetic interactions may potentiate the multifactorial liver damage induced mainly by PMRP through ABS administration.

Drug-induced liver injury (DILI) is clinically categorized as direct (dose-dependent, predictable), indirect (dose-independent, secondary to pharmacological action), or idiosyncratic (IDILI), among which, IDILI is particularly relevant to PMRP-associated hepatotoxicity due to its unpredictable, multifactorial, and dose-independent nature [21]. IDILI cases, although rare, account for a disproportionate share of acute liver failure and post-marketing drug withdrawals [22,23,24]. Emerging evidence indicates that genetic factors, such as polymorphisms within the human leukocyte antigen (HLA) region, as well as environmental factors, including comedications and infections, may modulate individual susceptibility to IDILI [25,26]. Nevertheless, the translation of these associative findings into predictive biomarkers remains elusive.

Current standards for hepatotoxicity assessment are evolving to incorporate emerging methodologies, including an advanced validation of the Roussel Uclaf Causality Assessment Method (RUCAM) [27], organoid-based models [28], and high-content screening techniques [29]. Yet these techniques harbor their own limitations; their high costs, the absence of an integrated metabolic environment, lack of standardized protocols, and poor interlaboratory reproducibility collectively hinder their application to mechanistic deep diving [30,31]. Consequently, a comprehensive approach is required to systematically correlate ABS constituents to their potential toxicological outcomes. Notably, PMR, the botanical precursor to PMRP, has been specifically listed in the dataset maintained by the U.S. National Library of Medicine [32], underscoring the urgency of this endeavor.

To address this gap, we developed a conceptual framework termed “PK screening coupled with dual-model verification.” This approach integrates three complementary elements: (1) comprehensive phytochemical profiling of ABS, (2) in vivo pharmacokinetic analysis in rats to establish systemic exposure levels of characteristic constituents and their metabolites, and (3) comparative hepatotoxicity evaluation using both normal and immune-stressed hepatocyte cell models to distinguish intrinsic toxicity from idiosyncratic susceptibility. Using this framework, we identified seven quality marker components in rat plasma following oral ABS administration via a validated UPLC–MS/MS method. The subsequent in vitro validation revealed, for the first time, that TSG, EM, liquiritigenin, and glycyrrhetinic acid exhibit idiosyncratic hepatotoxicity in hepatocytes at low concentrations, with EM retaining toxic activity at concentrations as low as 0.1 μM—a finding that conventional models, focused on acute high-dose exposure, would likely miss [33,34,35]. By transforming the current descriptive understanding of ABS-related hepatotoxicity into a mechanistic, component-resolved framework, this study not only identifies the material basis of PMRP-induced liver injury, but also provides a replicable methodological paradigm for evaluating the safety of complex herbal formulations. These findings offer actionable chemical insights for the rational clinical application of PMRP and ABS, bridging the critical gap between therapeutic efficacy and safety assessment.

## 2. Results

### 2.1. Quantitative Analysis of the Key Components in ABSs

Eight key components in the utilized ABSs were determined in the experiment (Figure S1). The linear calibration ranges for the determination of TSG, GLA, LI, EMG, and EM in ABS were Y = 6.74 × 10^3^X + 1.57 × 10^5^ (R^2^ = 0.9982, 1.0–2000 μg/mL), Y = 4.91 × 10^4^X − 5.62 × 10^3^ (R^2^ = 0.9998, 1.0–250 μg/mL), Y = 2.00 × 10^4^X − 7.21 × 10^3^ (R^2^ = 0.9990, 1.0–250 μg/mL), Y = 1.08 × 10^4^X − 1.84 × 10^3^ (R^2^ = 1.0000, 1.0–100 μg/mL), and Y = 6.40 × 10^4^X − 1.13 × 10^5^ (R^2^ = 0.9999, 0.5–250 μg/mL), respectively. The contents of IC, EP-C, and EP-B were determined using the methods outlined in the Chinese Pharmacopoeia, with IC as the reference standard, in which the corresponding correction factors were 1.00, 1.22, and 1.28, respectively. Accordingly, the contents of the eight key components are listed in Table 1.

### 2.2. Rat Plasma Pharmacokinetics of ABS with a Single Oral Administration

In addition to the eight major components of each TCM (IC, TSG, EP–C, EP–B, LI, GTA, EMG, and EM), certain representative metabolites (BA, 2’–O–RI II, and SA–B) were also included in the in vivo studies (Tables S1 and S2), thereby enabling the comprehensive identification of quality-representative key components through the amalgamation of in vivo and in vitro data.

**Figure 1:**
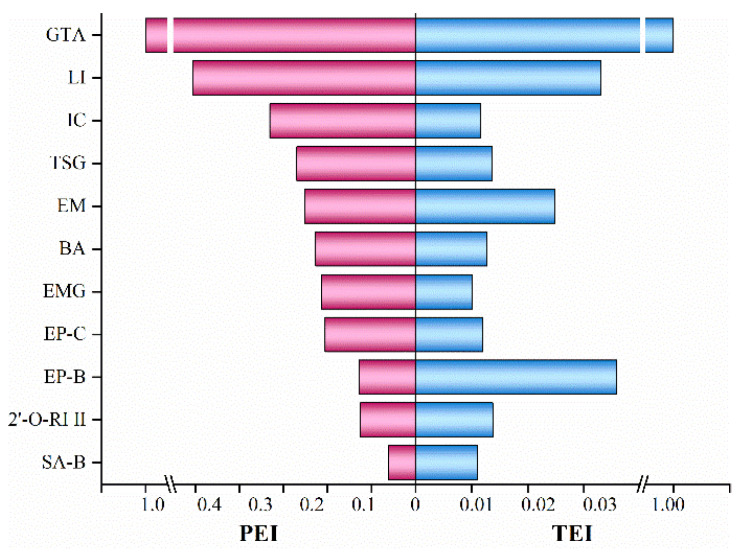
In vivo exposure levels of 11 candidate compounds in rat plasma following intragastric administration of the ABSs.

The experiment investigated the plasma exposure of eleven characteristic constituents of ABS, including several metabolites, in normal rats following a single intragastric administration at a volume of 10 mL/kg. To evaluate the in vivo exposure of these characteristic constituents, pharmacokinetic parameters were established in rats to evaluate the PEI and TEI, respectively (Figure 1). As a result, a set of seven representative quality markers of ABS was identified from eleven components, including IC, BA, TSG, LI, GTA, EMG, and EM. Their structures are presented in Figure S3.

**Figure 2:**
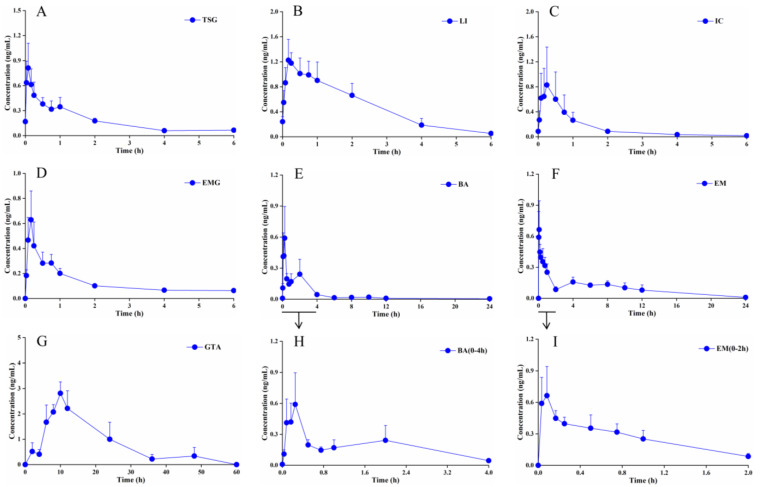
Mean plasma concentration–time profiles of the seven quality marker components following intragastric administration of 10 mL/kg ABS in rats (*n* = 6). (**A**) TSG. (**B**) LI. (**C**) IC. (**D**) EMG. (**E**) BA. (**F**) EM. (**G**) GTA. (**H**) BA (0–4 h). (**I**) EM (0–2 h).

The mean plasma concentration–time profiles of the seven ABS quality marker constituents after a single oral administration in rats are shown in Figure 2. The rat plasma pharmacokinetic parameters evaluated include the plasma peak concentration (C_max_) and the corresponding peak time (T_max_), the area under the plasma concentration–time curves (AUC_0–t_), and the terminal elimination half-life time (t_1/2_) for these main components found in vivo.

### 2.3. Sources and Attribution of the Principal Constituents in ABS

#### 2.3.1. Epimedii Folium: IC and Its Metabolites

IC represents the component with the highest mean content in ABSs, and BA is the primary metabolite of IC, formed via intestinal transformation. A secondary absorption phase of BA emerges after 4 h, corresponding to the point at which the plasma concentrations of IC were markedly reduced (Figure 2C,E).

#### 2.3.2. PMRP: TSG, EM, and EMG

The average content of TSG in ABS is second only to IC. Its T_max_ in ABS is approximately 6 min, indicating rapid absorption and entry into the systemic circulation. With a t_1/2_ of 2.46 h, TSG demonstrates the second-shortest elimination time among the seven representative quality markers, suggesting rapid metabolism in the body and a relatively short duration of pharmacological action (Table 2). EM exhibits a secondary plasma concentration peak approximately 2 h post-administration (Figure 2F). EMG shows near-total elimination from plasma after 2 h (Figure 2D).

#### 2.3.3. Glycyrrhizae Radix et Rhizome: GTA and LI

With the exception of GTA, the other six components attain peak blood concentrations within one hour after a single dose, indicating a relatively rapid absorption rate. In contrast, the T_max_ of GTA is 11 h, and its t1/2 is relatively prolonged at 8.70 h (Figure 2G). The AUC_0–t_ for LI is second only to that of GTA (Table 2).

### 2.4. Optimization of Concentration Range for the Target Compounds

**Figure 3:**
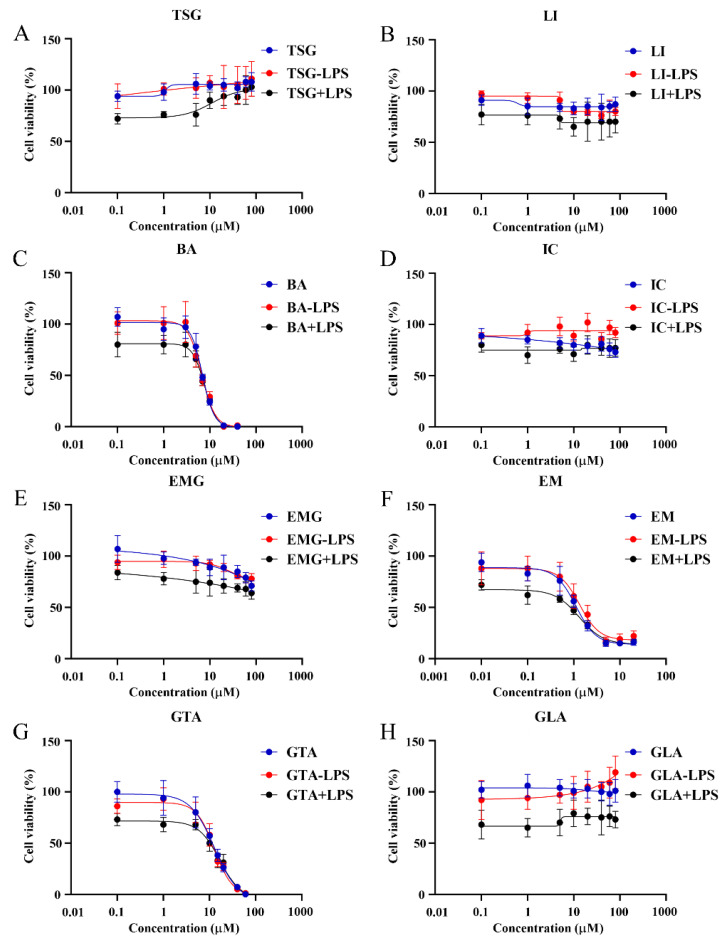
Drug concentration–cell viability curves of the seven ABS quality marker components and an original component of GTA in HepG2 cells (*n* = 6). (**A**) TSG. (**B**) LI. (**C**) BA. (**D**) IC. (**E**) EMG. (**F**) EM. (**G**) GTA. (**H**) GLA.

The study systematically evaluated the cytotoxicity of seven ABS quality markers and the original component GLA of GTA on HepG2 cells. The experimental design comprised three treatment groups: X, X − LPS, and X + LPS, with the X representing a set of eight distinct components. The X group represented the direct test group, whereas the X − LPS and X + LPS groups employed models involving co-administration with LPS. Specifically, in the X − LPS group, LPS was added to the blank control at a concentration of 10 μg/mL. Eight different substrate concentrations were prepared for the experiment (Figure 3). The concentrations were set as follows: EM at 0.01, 0.1, 0.5, 1, 2, 5, 10, and 20 μM; BA at 0.1, 1, 3, 5, 7, 10, 20, and 40 μM; GTA at 0.1, 1, 5, 10, 15, 20, 40, and 60 μM; and the remaining five components at 0.1, 1, 5, 10, 20, 40, 60, and 80 μM.

### 2.5. Hepatotoxic Effects of the Target Compounds in HepG2 Cells

**Figure 4:**
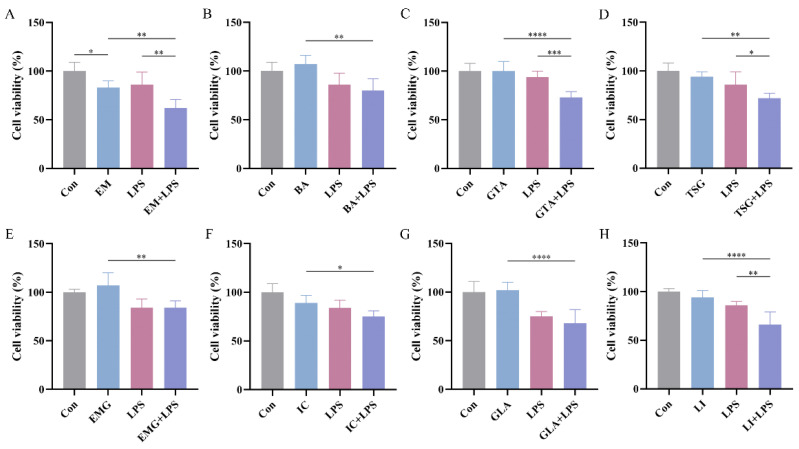
Hepatotoxicity assessment of seven ABS quality marker components and an original component of GTA in normal HepG2 cells and LPS-induced susceptible liver injury cells (*n* = 6). (**A**) EM. (**B**) BA. (**C**) GTA. (**D**) TSG. (**E**) EMG. (**F**) IC. (**G**) GLA. (**H**) LI. * *p* < 0.05, ** *p* < 0.01, *** *p* < 0.001, **** *p* < 0.0001.

**Figure 5:**
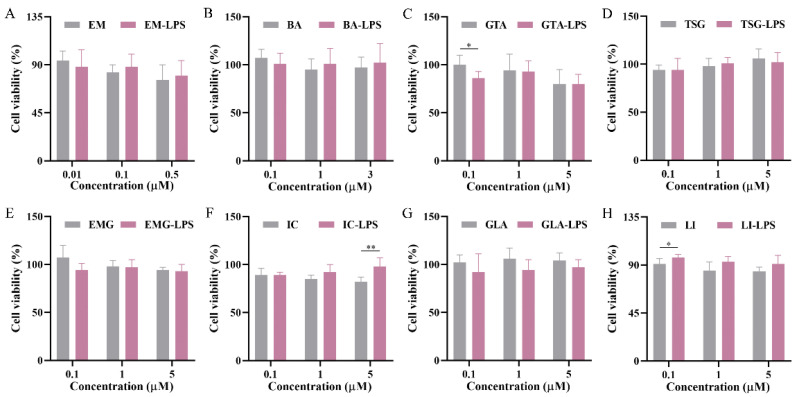
Effect of LPS on the hepatotoxicity of individual ABS quality marker components and GLA at low, medium, and high concentrations (*n* = 6). (**A**) EM. (**B**) BA. (**C**) GTA. (**D**) TSG. (**E**) EMG. (**F**) IC. (**G**) GLA. (**H**) LI. * *p* < 0.05, ** *p* < 0.01.

The half maximal inhibitory concentration (IC50) values of the eight target compounds were calculated from the concentration–cell viability curves. As shown in Figure 3, EM exhibits a low IC50 value of 1.12 Μm with 95% confidence intervals (CIs, 0.83–1.52 μM) in HepG2 cells. Additionally, BA and GTA demonstrate hepatotoxic effects, with IC50 values of 6.97 μM (95% CIs: 6.08–8.06 μM) and 12.8 Μm (95% CIs: 11.34–15.08 μM), respectively. EM, BA, and GTA exhibit significantly greater hepatotoxic potential compared to the other five substances under these in vitro conditions (Figure 3C,F,G). EMG, IC, and GLA exhibit relatively low inherent hepatotoxicity within the tested concentration range (0.1–80 μM) (Figure S4). TSG and LI are found to exhibit no hepatotoxic effects within the therapeutic concentration range. Notably, TSG confers a moderate hepatoprotective effect at high concentrations. However, it is observed that both TSG and LI elicit signs of hepatic damage in the LPS-induced immune stress hepatocyte model (Figure 4). Except for the 0.1 μM GTA, 5 μM IC, and 0.1 μM LI group, no statistically significant differences are observed between the X − LPS group and the X group for any of the other five substances at any concentration (*p* > 0.05) (Figure 5). In the X + LPS group, GTA demonstrates a significant inhibitory effect (*p* < 0.05). In contrast, both LI (*p* < 0.05) and the high-concentration of IC (*p* < 0.01) have exhibited synergistic effects by enhancing HepG2 cell viability.

### 2.6. Screening the Idiosyncratic Hepatotoxic Components of ABS

Given that low-concentration scenarios more accurately reflect clinical reality, they warrant particular emphasis and more detailed analyses. At a concentration of 0.1 μM, EM exhibits direct cytotoxicity toward normal cells (*p* < 0.05), and this toxicity has been further exacerbated under LPS-mediated cell susceptibility conditions (*p* < 0.01) (Figure 4A). In contrast to the absence of cytotoxicity on normal cells under the tested conditions, TSG, LI, and GTA demonstrate significant increased cytotoxicity in LPS-mediated susceptible cells with *p* < 0.05, *p* < 0.01, and *p* < 0.001, respectively (Figure 4C,D,H). BA, EMG, IC, and GLA exhibit no significant cytotoxicity in either normal or susceptible cells; however, statistically significant differences have been observed in their cytotoxicity toward these two cell types (*p* < 0.01, *p* < 0.01, *p* < 0.05, *p* < 0.0001, respectively) (Figure 4B,E,F,G). Their cytotoxicity is markedly amplified in susceptible cells.

## 3. Discussion

Studies have demonstrated that ABS effectively ameliorates cognitive dysfunction through the suppression of amyloid-β accumulation and phosphorylation, as well as Tau aggregation, collectively attenuating neuronal degeneration [1]. Additionally, it exhibits comparable or lower incidence rates of gastrointestinal disturbances and dizziness compared to Western medications or placebo controls [36]. As a result, ABS demonstrates substantial demand in clinical practice within China due to its consistent therapeutic efficacy. However, the proportion of PMRP is as high as 42.4% in the formulation of ABS, and its clinical usage has been associated with reported cases of abnormal liver function biochemical indicators [11,12]. Therefore, it is imperative to identify the hepatotoxic constituents of ABS and elucidate their metabolic profiles in vivo, thereby providing a scientific basis for rational drug administration and mitigating potential safety risks in clinical practice.

The quantitative analysis of the constituents in ABS indicates that IC exhibited the highest content of 234.5 μg/mL and served as the sole quality control marker for content determination specified in the Chinese Pharmacopoeia (no less than 60 μg/mL). The concentration of TSG ranked second, following closely behind that of IC. Therefore, from a content standpoint, the primary active ingredients of ABS are derived from *Epimedii Folium* and PMRP.

The pharmacokinetic profile of key bioactive constituents in TCMs has been utilized to predict their therapeutic efficacy and potential toxicological implications [37]. The PEI and TEI were employed to evaluate the in vivo exposure levels of individual components and their metabolites. The greater the values of PEI and TEI, the higher the in vivo exposure of the investigated constituents. Seven out of eleven components were screened based on their contents and exposure levels in rat plasma as the quality markers of ABS. Compared with the monomer [38], the intragastric administration of ABS resulted in a shortened t_1/2_ of IC, suggesting an accelerated elimination of IC from the body. This observation is consistent with the ability of certain components in ABS to induce the activity of metabolic enzymes, particularly the cytochrome P450 enzyme system, in the liver or intestine, thereby enhancing the rate of metabolic conversion [39,40]. Notwithstanding the various effects attributable to IC, such as tonifying kidney yang and dispelling wind dampness, the hepatotoxic risk associated with its principal metabolite, BA, lacks supporting biological data. It is therefore imperative to conduct cellular-level studies to clarify this risk.

TSG is the principal bioactive component of PMRP, demonstrating a spectrum of pharmacological activities such as anti-inflammatory, anti-aging, antioxidant, hepatoprotective, and antitumor effects [13,14]. PMRP is processed through the traditional method of “nine cycles of steaming and drying.” This process is arduous, requiring significant time and energy, and serves to attenuate toxicity while potentiating therapeutic efficacy, thereby transforming the properties of the raw PMR [41]. Although TSG possesses significant pharmacological activity, its safety profile, particularly regarding hepatotoxicity, has long been questioned, necessitating further conclusive research. The secondary plasma concentration peak of EM is attributed to fluctuations in blood drug levels caused by enterohepatic circulation and reabsorption [18,19]. Metabolic transformation represents another key contributing factor underlying the observed pharmacokinetic profile (Figure 2F). Conjugated anthraquinones like EMG are partially absorbed into the bloodstream in the small intestine, where they undergo hepatic metabolism to form free anthraquinones. The unabsorbed fraction undergoes microbial metabolism by colonic microbiota into free anthraquinones, thereby enabling subsequent absorption through the colonic wall [19,20]. These findings are in alignment with the observed elimination profile of EMG (Figure 2D). Given that EM is a potential factor of liver injury, and the level of EMG influences the final systemic exposure to EM undergoing metabolic transformation in vivo, rigorous quality control of EMG, a currently overlooked aspect, becomes essential.

LI and GTA are the principal constituents or major metabolites of *Glycyrrhizae Radix et Rhizome*, and they exhibit a broad spectrum of biological activities, including potent antioxidant, anti-inflammatory, antiviral, antitumor, and immunomodulatory activities [42]. The delayed pharmacokinetic profile of GTA is due to the fact that, under the influence of intestinal symbiotic bacteria, its precursor GLA is converted to the active metabolite GTA via the enzymatic hydrolysis and removal of two glucuronic acid molecules (Figure 2G).

Although the analytical method was validated per guidelines, the matrix effects for certain analytes were found to be between 50% and 60%. This may be attributed to the use of a single internal standard (1,8-dihydroxyanthraquinone) that is not structurally representative of all target compounds. While the accuracy and precision of the method remained acceptable, future studies should consider employing multiple class-specific internal standards (e.g., a stilbene glycoside analog for TSG; icariin-d3 or a similar flavonoid glycoside for Icariina) to better correct for matrix-induced ion suppression/enhancement.

In this study, the dual-model toxicity verification was used to screen the ABS active components related to their both direct and idiosyncratic hepatotoxic effects from high to low concentrations. The results suggest that BA, EM, and GTA exhibit direct cytotoxic effects and are considered to represent the substance basis for the hepatotoxicity of ABS (Figure 3C,F,G). Although EMG, IC, and GLA exhibit relatively low inherent hepatotoxicity, they are considered indirect hepatotoxicants through their metabolic conversion into EM, BA, and GTA in vivo [43,44,45], respectively, thereby representing non-negligible risk factors for liver injury. The hepatoprotective effect of TSG at high concentrations is consistent with the literature results [46]. The lack of significant differences between the X–LPS and X groups for most substances suggests that the interactions between these substances and LPS are negligible. The biological activities of the compounds vary considerably under immune stress. The synergistic hepatoprotective effects observed for IC and LI in the X + LPS group are consistent with their previously reported hepatoprotective effects [47,48].

Based on the analysis of hepatotoxic components at the relatively low concentrations, EM is the singular direct hepatotoxic component (DHC) (Figure 4A). Previous studies have suggested that mild immune stress (MIS) may be one of the susceptibility-related factors of IDILI caused by ABS [49]. In this paper, we found that the same dose of four components (TSG, EM, LI, and GTA) caused abnormal liver damage in MIS model cells, while it did not result in liver injury in normal cells, further confirming that MIS is a susceptibility factor for ABS-IDILI. This study is the first to categorize TSG, EM, LI, and GTA in ABS as idiosyncratic hepatotoxic components (IHCs). Notably, the TSG content in ABS is considerably high. Thus, uncovering its previously neglected risks related to IHCs carries substantial clinical importance. Furthermore, BA, EMG, IC, and GLA, while not cytotoxic in either condition, show amplified effects in susceptible cells, implicating their potential role as idiosyncratic hepatotoxicity susceptibility components (PIHSCs). Consequently, EM and TSG emerge, respectively, in ABS as the most typical DHC (at both high and low concentrations) and IHC, approaching clinical levels. This strategy will facilitate the accurate detection of diverse potential hepatotoxicity markers, which can then be applied to investigate the toxicological basis, pharmacological mechanisms, and therapeutic drug monitoring.

The findings could likely be generalized to other mammals because the identified toxic mechanisms (e.g., immune-stress response and metabolic pathways) are biologically conserved. However, direct quantitative extrapolation is limited by species differences in drug metabolism and gut microbiota. Under other experimental conditions, the findings are robust where immune activation is present, but generalizability to chronic use or multi-herb interactions requires further validation. Importantly, the study’s methodological framework is replicable for other herbal formulations. For human biology, the findings are highly relevant. They explain the clinical paradox of PMRP: its efficacy at safe doses yet unpredictable liver injury can occur in susceptible individuals. By identifying specific toxic components (e.g., EM at 0.1 μM) under immune-stressed conditions, the study provides a mechanistic basis for idiosyncratic hepatotoxicity and informs future clinical safety assessments.

## 4. Materials and Methods

### 4.1. Experimental Reagents

Chemical reference standards of TSG, EMG, EM, liquiritin (LI), icariin (IC), 18*β*–glycyrrhetinic acid (GTA), glycyrrhiic acid (GLA), epimedin B (EP–B), epimedin C (EP–C), 2′–*O*–rhamnosylicariside II (2′–*O*–RI II), and sagittatoside B (SA–B) were purchased from Shanghai Yuanye Biotech Co., Ltd. (Shanghai, China) with HPLC ≥ 98%. Baohuoside I (BA) and 1,8–dihydroxyanthraquinone (as the IS for the MS/MS determination) were purchased from Chengdu Pufei De Biotech Co., Ltd. (Chengdu, China) with HPLC ≥ 98%. HPLC-grade methanol was obtained from Tedia Company, Inc. (Fairfield, OH, USA). Lipopolysaccharide (LPS) (*Escherichia coli* 055:B5) was obtained from Sigma–Aldrich (Shanghai, China). The ABS (Lot: 2205656) was purchased from Jilin Aodong Pharmaceutical Group Co., Ltd. (Jilin, China), and the specification of ABS was 10 mL per bottle. The Cell Counting Kit–8 (CCK–8) was purchased from UElandy Biotechnology Co., Ltd. (Suzhou, China). Analytical-grade ethyl acetate and acetic acid were supplied by Nanjing Chemical Reagent Co., Ltd. (Nanjing, China). Ultrapure water was prepared using a Millipore purification system (Millipore, MA, USA).

### 4.2. Animals

Specific Pathogen-Free (SPF)-grade male Sprague–Dawley (SD) rats (6–8 weeks, 180–220 g) were purchased from Shanghai SIPPR–BK Laboratory Animal Co., Ltd. (License No.: SYXK 2023–0009). All rats were kept under SPF conditions: 25 ± 5 °C, 50 ± 20% humidity, 12/12 h light/dark cycle, with free access to food and water. All animal experiments were conducted in compliance with the Guide for the Care and Use of Laboratory Animals by the Chinese Association for Laboratory Animal Science and approved by the Animal Experiment Ethics Review Committee of China Pharmaceutical University (Ethical Approval No.: 202409132). The study was conducted and reported in accordance with the ARRIVE (Animal Research: Reporting of In Vivo Experiments) guidelines.

### 4.3. Pharmacokinetic Study

All rats underwent an acclimation period of a week and overnight fasting with free access to water only. Rats were randomly selected and administered the ABS (*n* = 6, biological replicates). Each rat was administered with a single ABS oral gavage dose of 10 mL/kg. Blood samples (approximately 150 μL) were collected from the orbital venous plexus using heparinized tubes at predefined time points (0, 0.033, 0.083, 0.167, 0.25, 0.5, 0.75, 1, 2, 4, 6, 8, 10, 12, 24, 36, 48, and 60 h) post-administration. The technical personnel were proficiently trained in performing orbital venous plexus blood draws, employing a technique designed to minimize discomfort to the animals. All rats received an intragastric administration of 1 mL saline at 6 h post-dosing, after which they were provided with unrestricted access to food and water for the remainder of the experimental period. The plasma samples were obtained through centrifugation of the bloods at 400 *g* force for 10 min at 4 °C and stored in Eppendorf tubes at −80 °C until analysis. An aliquot of 50 μL plasma was spiked with 10 μL of the internal standard (IS, 500 ng/mL), 0.2 mL saline, and 0.5 mL ethyl acetate. The mixture was vortexed for 5 min and subsequently centrifuged at 12,000 *g* force and 4 °C for 10 min to facilitate analyte extraction. The supernatant was carefully collected and evaporated to dryness at 40 °C using a ZLS–1 vacuum centrifugal concentrator. The residue was reconstituted with 100 μL of methanol–water solution (60:40, *v*/*v*) by vortex mixing for 5 min and centrifugation at 12,000 *g* force and 4 °C for 10 min. Then, a 10 μL aliquot of the resulting supernatant was injected into the UPLC–MS/MS system for the analysis.

The ABSs sourced from Jilin Aodong Pharmaceutical Group Co. Ltd. were utilized in the experiment. HPLC analysis for eight key components in the ABS was based on the previously reported method [12] and was performed at a flow rate of 1.0 mL/min, using acetonitrile and water containing 0.1% phosphoric acid as the mobile phases A and B, respectively. The elution program was set as follows (A:B): 0 min (10:90) → 10 min (20:80) → 20 min (30:70) → 40 min (45:55) → 55 min (90:10) → 59 min (90:10) → 60 min (10:90). PDA detection wavelength was 254 nm.

### 4.4. Evaluation of Plasma Exposure Level

The peak exposure indices (PEIs) and total exposure indices (TEIs) were calculated using the following formula, in which *n* represents each compound: PEI = $\bar{C}$_max (n)_/($\bar{C}$_max_) _max_, TEI = $\bar{AUC}$_(n)_/$\bar{AUC}$_max_. The quality markers of ABS were identified by considering the regression area (RA) of each compound after data normalization (RA ≥ 0.9 × $\bar{RA}$) based on their plasma exposure levels.

### 4.5. Plasma Sample UPLC–MS/MS Assay Parameters

An UPLC-MS/MS system (TSQ Quantis, Thermo Scientific, San Jose, CA, USA) equipped with a heated electrospray ionization interface (HESI) operating in the negative ion mode was used for the plasma sample analysis of IC, BA, TSG, EP–C, 2’–O–RI II, EP–B, SA–B, GTA, LI, EMG, and EM. Chromatographic separation was performed on a Thermo BDS Hypersil C_18_ column (100 × 4.6 mm, 3 µm) with linear gradient elution at a flow rate of 0.7 mL/min using methanol and water both containing 0.1% acetic acid as the mobile phases A and B, respectively. The gradient elution program was set as follows (A:B): 0 min (30:70) → 1 min (30:70) → 3 min (95:5) → 6.5 min (95:5) →7 min (30:70) → 8 min (30:70). The column temperature was maintained at 40 °C. Representative chromatograms of all 11 components were provided in Figure S2. The MS conditions were as follows: spray voltage of—4.5 KV; ion transfer tube temperature and vaporizer temperature of 350 °C; nitrogen sheath gas of 241 kPa; and auxiliary gas of 35 kPa. The quantification analysis was conducted under multiple reaction monitoring mode with argon collision gas pressure set at 0.2 Pa for collision-induced dissociation. The ion reactions were optimized as follows: *m*/*z* 721.5@18 eV → 513.1 for IC, *m*/*z* 513.0@26 eV → 366.0 for BA, *m*/*z* 405.3@19 eV → 243.0 for TSG, *m*/*z* 867.4@20 eV → 659.1 for EP–C, *m*/*z* 659.5@ 33 eV → 365.9 for 2’–O–RI II, *m*/*z* 853.5@18 eV → 645.1 for EP–B, *m*/*z* 645.5@34 eV → 366.0 for SA–B, *m*/*z* 469.5@38 eV → 425.1 for GTA, *m*/*z* 417.3@20 eV → 255.0 for LI, *m*/*z* 431.1@30 eV → 268.9 for EMG, *m*/*z* 269.0@20 eV → 225.0 for EM, and *m*/*z* 239.2@27 eV → 210.9 for IS.

### 4.6. Cell Culture

HepG2 cells were purchased from the Cell Bank of Chinese Academy of Sciences (Shanghai, China) and cultured in Roswell Park Memorial Institute (RPMI–1640) with 10% fetal bovine serum and 1% penicillin–streptomycin at 37 °C in a humidified atmosphere of 5% CO_2_. The culture medium was replenished at least every two days to sustain exponential growth.

### 4.7. Evaluation of Hepatotoxicity In Vitro

When conducting an experiment, cells were cultured overnight in a 96-well plate at 8 × 10^3^ cells/well, after which the inhibitory effects of seven target compounds (TSG, LI, BA, IC, EM, EMG, and GTA) and GLA, the original component of the metabolite GTA, on the proliferation of HepG2 cells were evaluated in a series of concentrations using the CCK–8 assay and *n* = 6 for the biological repetition. Simultaneously, additional experimental groups were established based on whether or not the control group was supplemented with LPS. A separate cohort of HepG2 cells was seeded into 96-well plates and cultured for 24 h. Thereafter, the cells were co-cultured with 10 μg/mL LPS and each of the eight target compounds at varying concentrations for 48 h. The culture medium was subsequently removed, and a 10% CCK–8 working solution was added to each well, followed by incubation for 1 h. The optical density (OD) at 450 nm for each well was measured using a microplate reader (Thermo Fisher, Waltham, MA, USA). Cell viability was calculated using the following formula: Cell viability (%) = (OD_sample_ − OD_blank_)/(OD_control_ − OD_blank_) × 100.

### 4.8. Statistical Analysis

WinNonlin software version 7.0 (Pharsight, St. Louis, MO, USA) was used to analyze pharmacokinetic parameters. The mean plasma concentration–time profiles of the representative components were plotted using Origin 2024b (OriginLab Corporation, Northampton, MA, USA), and the data are expressed as mean ± standard error. Statistical analysis was performed using GraphPad Prism 8.0 (GraphPad Software Inc., San Diego, CA, USA) to determine differences between two groups via a two-tailed Student’s *t*-test, and one-way ANOVA was used for three or more groups of data. *p* < 0.05 was considered statistically significant.

## 5. Conclusions

ABS is an OTC TCM formulation with very high annual consumption due to its remarkable therapeutic effects and widespread clinical indications. In this study, seven representative quality marker components of ABS have been identified leveraging UPLC–MS/MS, with three derived from PMRP. Furthermore, cellular-level hepatotoxicity assessments elucidate the substance basis underlying ABS-induced liver injury related with the relatively high dose level of PMRP. Unlike conventional toxicity evaluations that predominantly employ high-concentration exposures, our work has identified one DHC and four IHCs under low-concentration conditions approximating clinical dosage regimens. This study will provide a scientific foundation for the rational clinical application of ABS. However, the plasma concentrations of various components in rats remain relatively low and demonstrate significant inter-individual variability owing to limitations associated with the administration method, in which no concentration or lyophilization processes are applied to guarantee complete absorption of all constituents, including volatile compounds.

## Figures and Tables

**Table 1:** Concentrations of key components determined in the ABSs (*n* = 3).

Components	Concentrations (μg/mL)
IC	234.5 ± 0.2
TSG	232.0 ± 3.7
EP-C	28.8 ± 0.1
EP-B	34.7 ± 0.1
GLA	11.2 ± 0.1
LI	8.78 ± 0.07
EMG	4.81 ± 0.08
EM	3.00 ± 0.02

**Table 2:** Pharmacokinetic parameters of the seven representative constituents of ABS quality markers in rats after a single oral gavage dose of 10 mL/kg (*n* = 6).

Ingredients	C_max_	T_max_	AUC_0–t_	t_1/2_
	(ng/mL)	(h)	(h · ng/mL)	(h)
IC	1.08 ± 0.72	0.31 ± 0.11	0.90 ± 0.47	1.35 ± 0.24
BA	0.74 ± 0.29	0.82 ± 0.38	0.99 ± 0.28	7.58 ± 1.96
TSG	0.88 ± 0.27	0.10 ± 0.03	1.06 ± 0.20	2.46 ± 0.40
GTA	3.27 ± 0.17	11.0 ± 0.58	77.5 ± 1.43	8.70 ± 0.38
LI	1.65 ± 0.22	0.20 ± 0.03	2.52 ± 0.44	1.26 ± 0.09
EMG	0.70 ± 0.25	0.31 ± 0.14	0.78 ± 0.13	4.00 ± 1.35
EM	0.82 ± 0.25	0.44 ± 0.16	1.92 ± 0.36	4.63 ± 1.53

## Data Availability

The original contributions presented in the study are included in the article/Supplementary Material; further inquiries can be directed to the corresponding authors.

## Supplementary Materials

The following supporting information can be downloaded at: https://www.mdpi.com/article/10.3390/ph19030404/s1, Table S1. The linear range, regression data and LLOQ for the determination of eleven characteristic components in ABS for their plasma exposure evaluation by UPLC-MS/MS. Table S2. Summary of method validation results for eleven key components of ABS in rats’ plasma samples. Figure S1. HPLC–UV chromatograms of eight key components determined in the ABS. Figure S2. UPLC–MS/MS chromatograms of eleven components determined in the rats’ plasma samples. Figure S3. Chemical structures of the quality marker components associated with ABS. Figure S4. The primary metabolic pathways and corresponding metabolites of the three glucoside types or their analogs.

## Abbreviations

ABS	Anshenbunao syrup
BA	baohuoside I
DHC	direct hepatotoxic component
PMRP	Polygoni Multiflori Radix Praeparata
DILI	Drug-induced liver injury
EM	emodin
LI	liquiritin
TSG	2,3,5,4′–Tetrahydroxystilbene–2–O–β–D–glucoside
GTA	18β–glycyrrhetinic acid
PMR	Polygoni Multiflori Radix
TCM	traditional Chinese medicine
EMG	emodin–8–O–β–D–glucoside
HLA	human leukocyte antigen
EP–B	epimedin B
EP–C	epimedin C
2’–O–RI II	2’–O–rhamnosylicariside II
SA–B	sagittatoside B
HESI	heated electrospray ionization interface
OD	optical density
C_max_	plasma peak concentration
T_max_	corresponding peak time
AUC_0–t_	area under the plasma concentration–time curves
t_1/2_	terminal elimination half-life time
IHC	idiosyncratic hepatotoxic component
