# Supplementary material for: Hepatotoxicity Assessment of Anshenbunao Syrup by Multi-Component Quantification In Vivo/In Vitro and Cell Biological Evaluations
Source: Pharmaceuticals (Basel). 2026 Mar 1;19(3):404. doi: 10.3390/ph19030404 (PMC13028611; doi:10.3390/ph19030404)
Supplement: Supplementary file 1 [file pharmaceuticals-19-00404-s001.zip › pharmaceuticals-4150480-supplementary.pdf]

# Supporting information

## Hepatotoxicity Assessment of Anshenbunao Syrup by Multi-Component Quantification In Vivo/In Vitro and Cell Biological Evaluations

Lan Chen [1], Zhizhen Wei [1], Rui Cheng [1], Pengwei Hu [1], Shixiao Wang [1], Wei Wu [2], Adouani Imene [3], Yuan Zhang [4], Fengming Chen [5,*] and Taijun Hang [1,*]

1 Department of Pharmaceutical Analysis, China Pharmaceutical University, Nanjing 210009, China; cl2721401467@163.com (L.C.); wzz2268209408@163.com (Z.W.); chengrui123ing@163.com (R.C.); hpw18851897876@163.com (P.H.); wangsx_yes@163.com (S.W.)

2 School of Pharmacy, Shandong Second Medical University, Weifang 261053, China; wuwei@sdsmu.edu.cn

3 Department of Pharmacy, Faculty of Medicine, University of Ferhat Abbas Setif 1, Setif 19000, Algeria; lill_yen@hotmail.fr

4 School of Pharmacy, China Medical University, Shenyang 110122, China; 13840149878@163.com

5 Institute of Food Safety, Chinese Academy of Quality and Inspection & Testing, Beijing 100176, China.

* Correspondence: chenfengmingok@163.com (F.C.); hangtj@cpu.edu.cn (T.H.); Tel.: +86-010-53898026 (F.C.); +86-025-86185555 (T.H.)

**Table of contents:**

Table S1. The linear range, regression data and LLOQ for the determination of eleven characteristic components in ABS for their plasma exposure evaluation by UPLC-MS/MS.

Table S2 Summary of method validation results for eleven key components of ABS in rats' plasma samples.

Figure S1. HPLC–UV chromatograms of eight key components determined in the ABS.

Figure S2. UPLC–MS/MS chromatograms of eleven components determined in the rats' plasma samples.

Figure S3. Chemical structures of the quality marker components associated with ABS.

Figure S4. The primary metabolic pathways and corresponding metabolites of the three glucoside types or their analogs.

**Table S1**. The linear range, regression data and LLOQ for the determination of eleven characteristic components in ABS for their plasma exposure evaluation by UPLC-MS/MS.

| Components | Regression equation | Linear range (ng/mL) | $R^2$ | LLOQ (ng/mL) |
|---|---|---|---|---|
| IC | $Y=1.89\times10^{-2}X-5.36\times10^{-4}$ | 0.05-100 | 0.9921 | 0.05 |
| BA | $Y=5.27\times10^{-2}X-5.02\times10^{-4}$ | 0.05-100 | 0.9909 | 0.05 |
| TSG | $Y=4.43\times10^{-2}X-3.89\times10^{-4}$ | 0.05-100 | 0.9904 | 0.05 |
| EP-C | $Y=5.34\times10^{-2}X-1.97\times10^{-3}$ | 0.05-100 | 0.9926 | 0.05 |
| 2'-O-RI II | $Y=1.53\times10^{-2}X-4.34\times10^{-4}$ | 0.05-100 | 0.9807 | 0.05 |
| EP-B | $Y=3.83\times10^{-3}X-1.75\times10^{-3}$ | 0.20-100 | 0.9859 | 0.20 |
| SA-B | $Y=1.87\times10^{-2}X-6.00\times10^{-4}$ | 0.05-100 | 0.9936 | 0.05 |
| GTA | $Y=8.77\times10^{-3}X-1.11\times10^{-3}$ | 0.10-100 | 0.9891 | 0.10 |
| LI | $Y=5.75\times10^{-2}X-7.27\times10^{-3}$ | 0.05-100 | 0.9907 | 0.05 |
| EMG | $Y=1.29\times10^{-1}X-4.81\times10^{-4}$ | 0.05-100 | 0.9916 | 0.05 |
| EM | $Y=3.40\times10^{-1}X-2.77\times10^{-1}$ | 0.02-100 | 0.9889 | 0.02 |

**Table S2**. Summary of method validation results for eleven key components of ABS in rats' plasma samples (n=6).

| Components | Concentration | Intra-batch (n=6) | | Inter-batch (n=3) | | Matrix effect (%) | Extraction recovery (%) |
|---|---|---|---|---|---|---|---|
| | (ng/mL) | RSD (%) | RE (%) | RSD (%) | RE (%) | Mean±SD, RSD | Mean±SD, RSD |
| IC | 0.1 | 9.68 | -4.86 | 10.17 | 7.76 | 60.20±7.46,12.39 | 87.97±9.62,10.94 |
| | 2 | 9.42 | -6.60 | 7.51 | -6.93 | 63.14±4.87, 7.72 | 79.71±7.78, 9.76 |
| | 20 | 8.40 | -9.61 | 8.46 | -7.33 | 61.69±3.21, 5.20 | 71.93±4.53, 6.30 |
| | 80 | 6.18 | -4.94 | 7.36 | -7.38 | 69.52±5.39, 7.75 | 86.15±3.72, 4.32 |
| BA | 0.1 | 11.77 | 0.78 | 12.79 | -2.37 | 54.92±3.22, 5.86 | 74.58±4.10, 5.50 |
| | 2 | 9.39 | -6.52 | 10.38 | -8.36 | 58.86±3.50, 5.94 | 79.75±7.12, 8.93 |
| | 20 | 9.53 | -0.31 | 9.55 | 5.55 | 62.25±3.56, 5.73 | 69.32±6.17, 8.90 |
| | 80 | 6.43 | -3.65 | 8.37 | -6.61 | 79.23±3.22, 4.07 | 65.97±1.24, 1.88 |
| TSG | 0.1 | 9.59 | 8.94 | 8.56 | 9.96 | 64.56±2.99, 4.63 | 87.69±11.96,13.64 |
| | 2 | 8.07 | 4.54 | 8.77 | 7.14 | 72.54±2.28, 3.14 | 87.11±8.82,10.13 |
| | 20 | 8.24 | -3.60 | 8.85 | 3.90 | 72.89±5.70, 7.82 | 81.80±6.10, 7.45 |
| | 80 | 8.18 | -3.01 | 8.09 | -3.39 | 67.74±5.30, 7.83 | 80.55±6.41, 7.95 |
| EP-C | 0.1 | 10.62 | -3.14 | 9.75 | 5.53 | 64.95±7.31,11.26 | 63.91±4.15, 6.50 |
| | 2 | 8.71 | 5.05 | 9.40 | -7.17 | 81.08±6.32, 7.79 | 91.14±4.25, 4.66 |
| | 20 | 9.76 | -7.60 | 8.44 | -6.76 | 55.47±4.80, 8.66 | 93.58±6.73, 7.19 |
| | 80 | 8.23 | -8.77 | 8.81 | -8.97 | 64.14±6.27, 9.77 | 98.91±5.93, 6.00 |
| 2'-O-RI Ⅱ | 0.1 | 6.22 | 1.12 | 7.27 | 7.27 | 70.09±5.50, 7.84 | 69.30±8.59,12.39 |
| | 2 | 7.59 | -7.57 | 9.66 | -4.50 | 65.40±6.06, 9.26 | 78.37±3.80, 4.85 |
| | 20 | 5.69 | -5.88 | 9.79 | -1.94 | 55.26±4.48, 8.11 | 60.72±3.31, 5.45 |
| | 80 | 5.95 | -4.05 | 9.11 | -7.02 | 67.51±6.58, 9.74 | 72.17±6.51, 9.01 |
| EP-B | 0.1 | 11.66 | -9.51 | 9.61 | 1.07 | 60.40±7.41,12.27 | 75.56±7.34, 9.71 |
| | 2 | 7.90 | -7.37 | 9.23 | -9.36 | 54.07±3.07,5.67 | 85.66±7.23, 8.45 |
| | 20 | 7.69 | -9.81 | 8.68 | -4.12 | 57.88±4.38, 7.57 | 81.53±3.89, 4.78 |
| | 80 | 7.61 | -7.78 | 8.88 | -8.19 | 55.98±2.64, 4.71 | 87.05±4.45, 5.11 |
| SA-B | 0.1 | 7.97 | -7.09 | 9.52 | -8.02 | 51.45±4.98, 9.68 | 81.96±4.65, 5.68 |
| | 2 | 8.76 | -9.04 | 9.28 | 7.39 | 51.92±3.13, 6.04 | 79.19±5.27, 6.65 |
| | 20 | 8.53 | 9.59 | 8.60 | 9.49 | 51.84±4.45, 8.58 | 75.72±4.50, 5.94 |
| | 80 | 6.66 | -5.76 | 4.87 | -6.93 | 61.26±2.80, 4.57 | 96.50±9.56, 9.91 |
| GTA | 0.1 | 10.28 | -7.00 | 10.32 | -8.96 | 77.19±7.65, 9.92 | 90.61±8.46, 9.34 |
| | 2 | 9.97 | 8.42 | 9.16 | 7.49 | 59.46±5.59, 9.40 | 81.92±7.81, 9.53 |
| | 20 | 9.71 | -4.62 | 8.78 | 7.59 | 55.30±2.26, 4.09 | 76.82±5.87, 7.64 |
| | 80 | 9.63 | -4.67 | 3.04 | 4.07 | 58.61±3.13, 5.34 | 83.24±3.49, 4.20 |
| LI | 0.1 | 8.08 | 7.83 | 9.61 | -5.31 | 59.17±2.72, 4.59 | 70.27±10.13,14.41 |
| | 2 | 9.95 | -5.50 | 10.65 | 1.40 | 63.61±5.16, 8.12 | 91.10±8.91, 9.78 |
| | 20 | 8.86 | -5.78 | 9.08 | -2.89 | 54.56±4.30, 7.89 | 73.05±7.22, 9.88 |
| | 80 | 5.47 | -5.26 | 6.41 | -7.03 | 56.54±5.59, 9.88 | 107.37±9.91, 9.23 |

| | | | | | | | |
|---|---|---|---|---|---|---|---|
| EMG | 0.1 | 11.75 | 0.16 | 11.57 | -3.24 | 53.95±3.76, 6.96 | 82.09±8.83,10.76 |
| | 2 | 11.03 | 7.42 | 12.30 | 6.60 | 61.43±5.70, 9.29 | 82.66±2.36, 2.86 |
| | 20 | 12.99 | -7.52 | 12.31 | -7.76 | 49.92±4.27, 8.55 | 74.69±2.07, 2.78 |
| | 80 | 10.78 | -3.66 | 11.62 | -7.82 | 60.65±5.20, 8.57 | 86.45±3.17, 3.67 |
| EM | 0.1 | 13.71 | 0.11 | 12.98 | -9.70 | 79.67±3.92, 4.92 | 82.82±9.86,11.90 |
| | 2 | 12.96 | -2.80 | 12.85 | -6.01 | 79.72±4.99, 6.26 | 85.58±8.03, 9.39 |
| | 20 | 7.97 | -4.01 | 12.53 | -7.47 | 79.75±7.50, 9.41 | 78.61±1.77, 2.25 |
| | 80 | 8.49 | -6.04 | 9.16 | -6.33 | 61.86±4.97, 8.03 | 88.76±7.33, 8.25 |

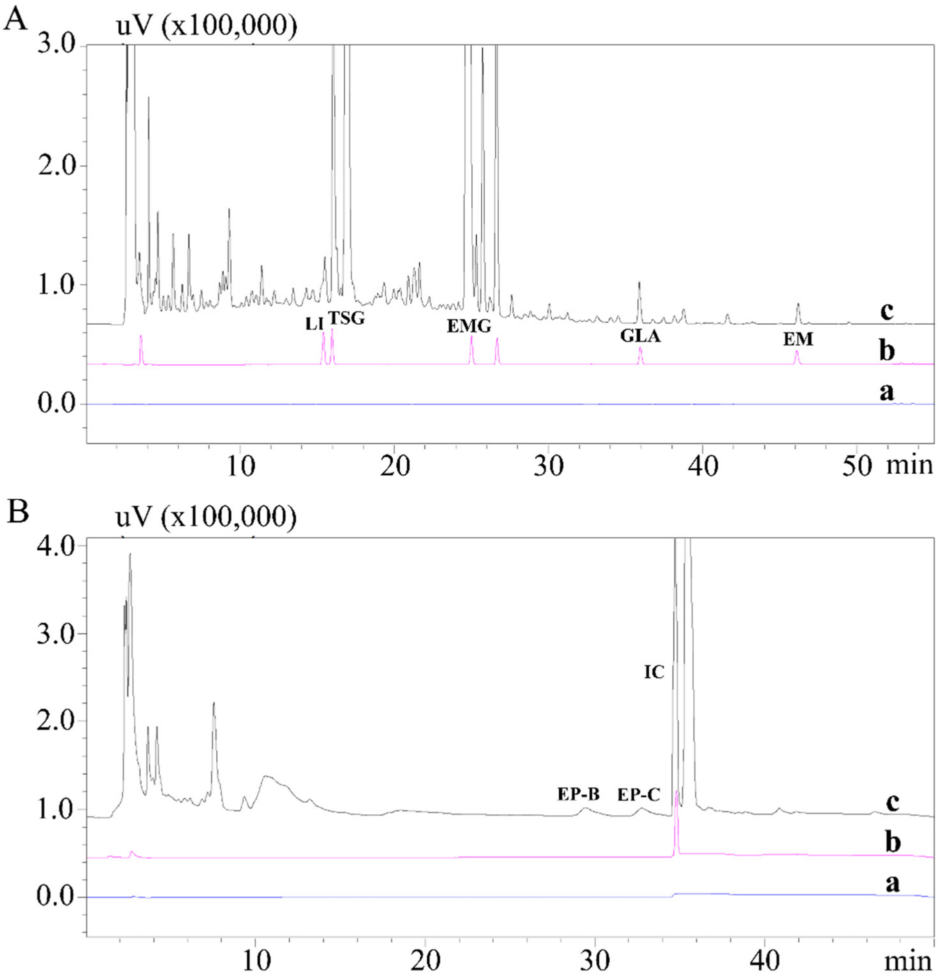


Figure S1. HPLC–UV chromatograms of eight key components determined in the ABS. (**A**). external standard method: a. blank, b. reference mixture containing 10 μg/mL LI, 10 μg/mL TSG, 2 μg/mL EMG, 8 μg/mL GLA, and 2 μg/mL EM, c. commercially available ABS. (**B**). quantitative analysis of multi–components by the single–marker (QAMS) method: a. blank, b. reference of 40 μg/mL IC, c. commercially available ABS.

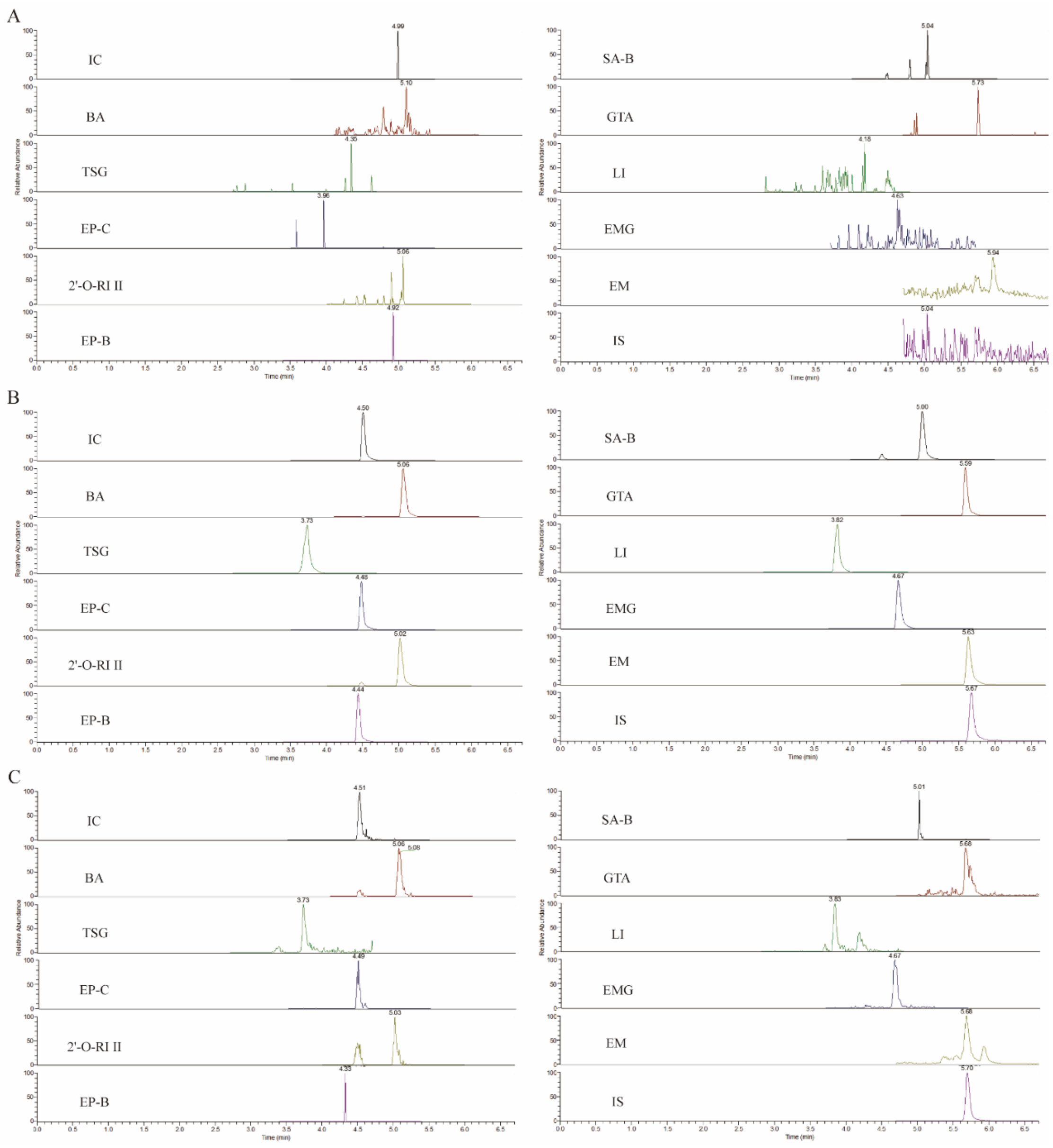


Figure S2. UPLC–MS/MS chromatograms of eleven components determined in the rats' plasma samples. (**A**) blank plasma. (**B**) blank plasma spiked with IC, BA, TSG, EP-C, 2'-O-RI II, EP-B, SA-B, GTA, LI, EMG, EM, and IS. (**C**) plasma samples obtained from rats after oral administration of ABS.

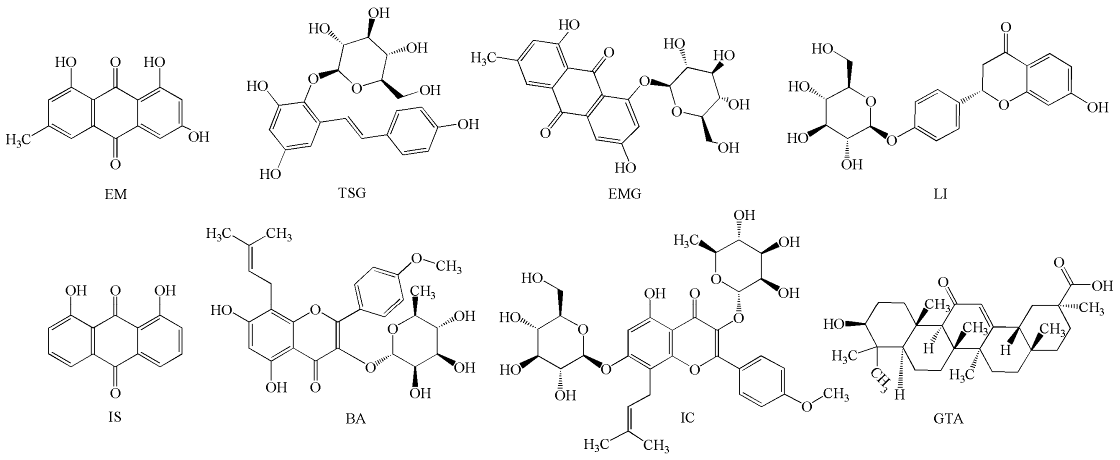


Figure S3. Chemical structures of the quality marker components in ABS.

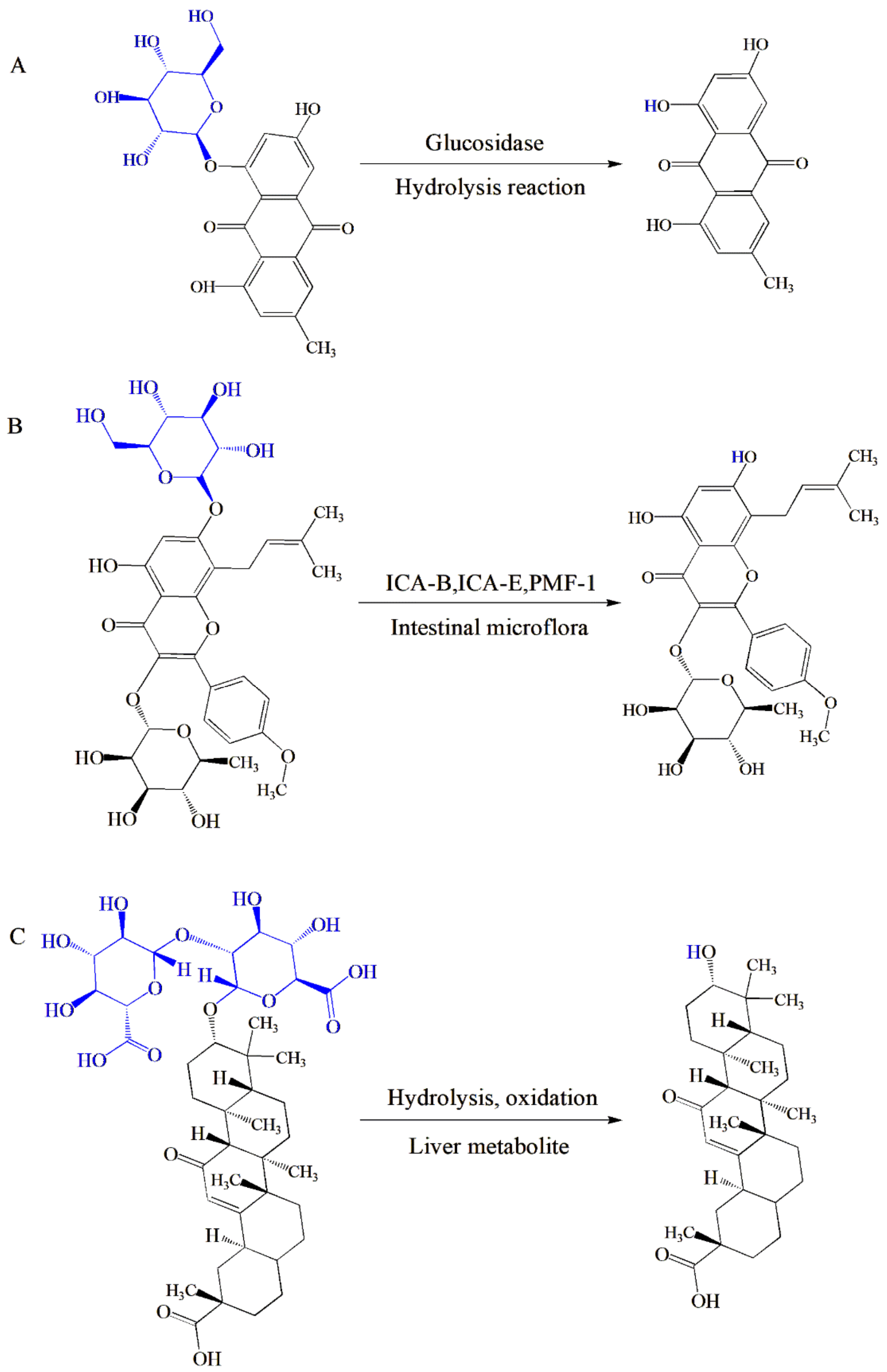

Figure S4. The primary metabolic pathways and corresponding metabolites of the three glucoside types or their analogs. (**A**) EMG. (**B**) IC. (**C**) GLA.
